# A systematic overview of prospective cohort studies of cardiovascular disease in sub-Saharan Africa

**DOI:** 10.5830/CVJA-2011-042

**Published:** 2012-03

**Authors:** Andre Pascal Kengne, Lucas M Ntyintyane, Bongani M Mayosi

**Affiliations:** The George Institute for International Health, University of Sydney, Sydney, Australia; Department of Medicine, Groote Schuur Hospital and University of Cape Town, Cape Town, South Africa; National Collaborative Research Programme on Cardiovascular and Metabolic Disease, Medical Research Council, Cape Town, South Africa; Department of Medicine, Groote Schuur Hospital and University of Cape Town, Cape Town, South Africa; Department of Medicine, Groote Schuur Hospital and University of Cape Town, Cape Town, South Africa

**Keywords:** cohort studies, cardiovascular diseases, risk factors, outcomes, sub-Saharan Africa

## Abstract

**Background:**

Cardiovascular diseases (CVDs) are becoming increasingly significant in sub-Saharan Africa (SSA). Reliable measures of the contribution of major determinants are essential for informing health services and policy solutions.

**Objective:**

To perform a systematic review of all longitudinal studies of CVDs and related risk factors that have been conducted in SSA.

**Data source:**

We searched electronic databases from 1966 to October 2009. Published studies were retrieved from PubMed and Africa EBSCO. Reference lists of identified articles were scanned for additional publications.

**Study selection:**

Any longitudinal study with data collection at baseline on major cardiovascular risk factors or CVD, including 30 or more participants, and with at least six months of follow up were included.

**Data extraction:**

Data were extracted on the country of study, year of inception, baseline evaluation, primary focus of the study, outcomes, and number of participants at baseline and final evaluation.

**Results:**

Eighty-one publications relating to 41 studies from 11 SSA countries with a wide range of participants were included. Twenty-two were historical/prospective hospital-based studies. These studies focused on risk factors, particularly diabetes mellitus and hypertension, or CVD including stroke, heart failure and rheumatic heart disease. The rate of participants followed through the whole duration of studies was 72% (64–80%), with a significant heterogeneity between studies (for heterogeneity, *p* < 0.001). Outcomes monitored during follow up included trajectories of risk markers and mortality.

**Conclusions:**

Well-designed prospective cohort studies are needed to inform and update our knowledge regarding the epidemiology CVDs and their interactions with known risk factors in the context of common infectious diseases in this region.

## Abstract

The pattern of disease occurrence in sub-Saharan Africa (SSA) is changing constantly, both at the level of and within broad categories of disease entities. Over the past few decades, the significance of chronic diseases and principally cardiovascular diseases (CVD) has grown consistently in SSA. Within the broad category of cardiovascular diseases, a double burden of infectious and post-infectious diseases (i.e. rheumatic valve disease, post-tuberculosis cor pulmonale and pericardial tuberculosis) co-exists, with a rising burden of hypertension and its related complications of stroke, heart failure and chronic kidney disease.^1^-^3^

According to the global burden of disease estimates,^4^ in 2001, cerebrovascular diseases and ischaemic heart diseases (IHD) were the eighth and ninth leading causes of death in SSA, and contributed 3.3 and 3.2%, respectively, of total deaths recorded in that year. Overall, in 2001, 10% of all deaths in SSA occurred as a result of CVD, and 4% of disability-adjusted life years (DALYs) were related to a CVD. CVDs and chronic diseases are compounding an under-resourced and understaffed public care system in SSA, and there is a huge financial burden as well. SSA is a poor region with major socio-economic challenges. Projections indicate that by 2030, IHD and cerebrovascular diseases will overtake HIV/AIDS as the leading causes of death in this region. By then, the two CVD constituents will contribute over 20% of total deaths and 7% of DALYs in SSA. Diabetes mellitus will feature among the top 10 leading causes of death.^5^

A short window of opportunity still exists, during which it might be possible to introduce measures that would prevent the full development of this epidemic of cardiovascular diseases in SSA. Reliable information about the distribution of known risk factors, how they change with time and how they relate to cardiovascular outcomes is of major importance but still lacking in Africa.^6^ Without such reliable data it is impossible to devise effective, long-term disease-prevention strategies to combat the double burden.

Poor record keeping precludes the use of administrative databases to inform public healthcare policies. Cross-sectional data relating to the distribution of risk factors and the prevalence of CVD exist in some places, as summarised elsewhere.^1^,^7^-^12^ That the availability of this type of data has not produced the expected change in policies to counter the trend of CVD, highlights the need for more sensitive evidence on the ill effects of CVD in SSA. In the West for example, the observed decline in incidence of CVD has been largely influenced by evidence generated from longitudinal studies (interventional or not), including the landmark Framingham Heart Study initiated around the peak in incidence of CVD in that part of the world.

Longitudinal studies of cardiovascular diseases in Africa have several applications, including: (1) generating more sensitive information in the form of causal associations between risk factors and hard outcomes such as death and disability, and therefore increasing awareness and need for action; (2) contextualising the knowledge generated elsewhere on CVD, and accordingly, improving the local uptake of measures with proven benefits on cardiovascular outcomes in other parts of the world; (3) providing the unique opportunity of accurately characterising the early phases of epidemiological transition, and the interaction between CVD and prevalent infectious diseases; (4) providing local epidemiological training laboratories to mould the careers of many young African researchers to continue the fight against CVD across the generations; and (5) providing resources for collaboration between African researchers and their peers with similar interests around the world.

The study aim was to conduct a systematic review of the literature for all prospective cohort studies of cardiovascular traits that have measured exposure before outcome in SSA. We were interested in identifying gaps in the knowledge on the epidemiology of CVD in SSA. The objective was to assess the suitability of the available studies for reliably addressing research uncertainties through data pooling. Such information is useful for informing the immediate health services and policy solutions, and assisting the design and planning of relevant studies that will inform future strategies.

## Methods

## Data source

We systematically searched the PubMed and Africa EBSCO databases, using a strategy that included all possible combinations of three levels of medical subject heading terms: (1) ‘Africa south of the Sahara’ (2) ‘cohort studies’, ‘longitudinal studies’, ‘retrospective studies’, ‘prospective studies’, and (3) ‘cardiovascular diseases’, ‘stroke’, ‘hypertension’, ‘diabetes mellitus’, ‘smoking’ and ‘cholesterol’. The search was limited to studies in humans. The starting date from which articles were identified was from 1966 until October 2009.

We searched the database of cohort studies of the *International Journal of Epidemiology*. References quoted in original publications, two editions of a book on causes of deaths and diseases in Africa,^13^,^14^ and the INDEPTH network website^15^ were searched for additional information. The Global Cardiovascular InfoBase of the World Health Organisation^16^ was also consulted. We limited the review to articles that provided at least an abstract in the English language. Titles of the articles and abstracts were reviewed and relevant articles obtained if required. When the full article was needed and was not available to us, attempts were made to get one from the corresponding authors. References were extracted and stored with the use of Endnote V9.0.0 software (Thomson/ISI ResearchSoft, Berkeley, CA).

## Data selection

Two reviewers (APK and LNM) independently screened the articles for eligibility. The inclusion criteria were: prospective cohort design; measurement of exposure before outcome; minimum duration of follow up of six months; baseline assessment for at least one major risk factor other than gender and age (i.e. blood pressure variables/status for hypertension, lipid variables/status for dyslipidaemia, glucose exposure/status for diabetes, smoking status) or for a status for cardiovascular disease; and/or outcomes ascertainment during follow up, including trajectories of risk factors and mortality; and studies conducted in a sub-Saharan African country. We excluded migrant studies, studies with a focus on non-cardiovascular diseases, post-surgical intervention cohorts, and post-cardiac instrumentation cohorts. Cohorts with less than 30 participants at baseline were also excluded.

## Data extraction

We extracted data on the country of the study, the year of inception, main focus of the study, number of participants at baseline and final evaluation, the setting of the study (hospital, community, both, other), baseline measurement and outcomes, and the overall duration of follow up. We did not perform a quality assessment.

## Statistical analysis

To assess the homogeneity between studies, we computed the ratio of number of participants successfully traced at the final visit/number of participants assessed at baseline (with the accompanying 95% confidence intervals) for each study that provided enough data to compute this ratio. We then constructed a forest plot of these ratios and the pooled estimate, assuming a random effect model. These analyses were performed using the Comprehensive Meta Analysis V 2.2.046 (Biostat, Inc. Englewood, USA) and Meta-analysis with Interactive Explanations (MIX)^17^,^18^ V 1.7.

## Results

**Fig. 1:**
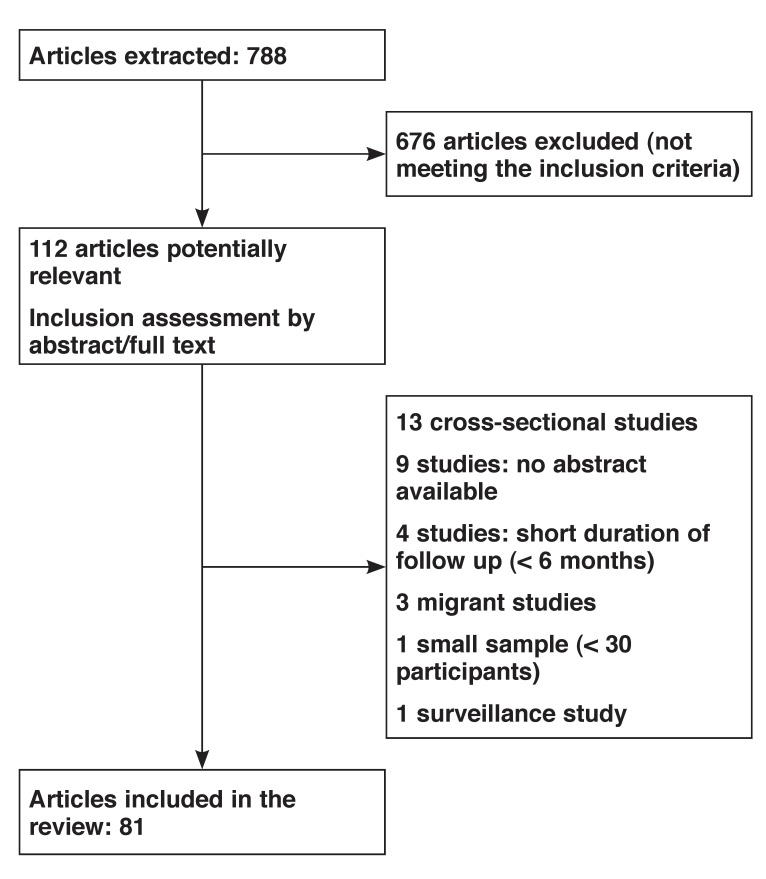
Flow chart of studies in the review.

The initial search of electronic databases revealed 788 entries published between 1966 and 2009. Of these references, 676 were excluded because they were not relevant to the purpose of this systematic review. A total of 81 references reporting on 41 studies were included in the final review (Fig. 1). These studies had been conducted in 11 sub-Saharan African countries, with about 59% of them in South Africa and Nigeria.

Articles relating to the same study were grouped and checked for consistency. Studies were hospital-based historical or prospective cohorts (22 studies) or community-based cohorts (10 studies). Few had a hospital and community component and work place-based cohorts (two studies). Few studies were still ongoing and others were conducted over a range of duration from six months to over 20 years.

The focus of these studies varied substantially, with a concentration however on CVD constituents and major risk factors such as hypertension and diabetes mellitus. Baseline evaluation also included either risk factors or status for specific cardiovascular diseases (Table 1). Outcomes monitored in general had a focus on mortality and trajectories of risk markers. The capacity of these studies for retaining participants during follow up showed some heterogeneity not explained by the duration of follow up or time period of the study (Table 2).

**Table 1:** Characteristics Of Included Studies

*Country, year of inception and reference*	*Population size*	*Main focus*	*Baseline evaluation*	*Settings*	*Duration of follow up*	*CVD outcomes*
Ethiopia 1983^46^	150	Post-stroke	Status for stroke	Hospital	2 years	Mortality
Ethiopia 1988^34,35,47^	1699	Diabetes mellitus	Status for diabetes	Hospital		Mortality
Gambia 1990^44^	106	Post stroke	Status for stroke	Hospital	4 years	Mortality
Ghana 1973^48^	155	Hypertension	BP	Hospital	1 year	Compliance to treatment
Kenya 1983^41,49-51^	592	BP	BP, BMI electrolytes	Community	2 years	Change in BP
Nigeria^40^	300	BP in pregnancy	Blood pressure	Hospital	9 months	Change in BP
Nigeria^26^	92	Rheumatic heart disease	Cardiac status	Hospital	10 years	Death complications
Nigeria^52^	107	Heart failure	Cardiac status, risk factors	Hospital	1 year	Mortality
Nigeria 1969–1972^23,28,53-55^	227	Peripartum cardiac failure	Status for heart failure	Hospital	25 years	Mortality, change in BP
Nigeria 1993^19,56,57^	4333	Adult mortality	Risk factors	Community	5 years	All-cause mortality
Nigeria 1993^58^	708	Post-stroke	Status at diagnosis	Hospital	6 months	Mortality
Nigeria 1995^36,59,60^	1344	BP and relative weight	BP and weight	Community	7 years	Change in risk factors, deaths
Senegal^33^	886	Hypertension in pregnancy	Hypertension	Hospital	9 months	Pregnancy outcome
Senegal 2003^61,62^	170	Post-stroke	Status at diagnosis	Hospital	1 year	Outcomes
South Africa, Nigeria, Cameroon 2004^6,37^	185	Pericardial tuberculosis	Cardiac status, status for chest and HIV infection	Hospital	6–12 months	Outcomes under treatment
South Africa 1972^30^	49	Familial hypercholesterolaemia	Familial hypercholesterolaemia	Hospital	13 years	Cardiovascular complications
South Africa 1970^43,63-67^	4925	Health of gold miners	Risk factors	Workplace (gold mines)	20 years	Mortality
South Africa 2006^68^		CVD and risk factors	Heart conditions and risk factors	Community/hospital	Ongoing	Incidence and outcome
South Africa^24^	210	Post-stroke	Functional status	Hospital	2 years	Mortality and disability
South Africa 1986^25^	711	Cardiac rehabilitation	N/A	Hospital	1.5 years	Dropout rate
South Africa 1966^69^	62	Diabetes mellitus	Risk factors/kidney functions	Hospital	12 years	Renal outcomes
South Africa 1965^31^	266	Glucose tolerance	Glucose tolerance status	Community	5 years	Incidence of diabetes
South Africa 1972^22^	168	Rheumatic heart disease	Cardiac sounds	School based	4 years	Evolution of the cardiac murmurs
South Africa 1979^70-85^	6332	Cardiovascular diseases	Risk factors	Community	4 years	Change in risk factors
South Africa 1982^29,86^	88	Type 1 diabetes	Diabetes status	Hospital	20 years	Mortality and complications
South Africa 1984^39,45,87^	2479	Glucose tolerance	Glucose tolerance status	Community	10 years	Incidence of diabetes
South Africa 1989^88-90^	3273	Epidemiological transitions	Risk factors	Community/Hospital	Ongoing	Change in risk factors
South Africa 1992^91,92^	3147	Hypertension	BP	Hospital	1 year	Death, control, compliance
South Africa 1996^93-98^	1884	Children, growth and health	Anthropometric and BP	Community/Schools	Ongoing	Change in BP and anthropometric measurements
South Africa 1999^21^	200	Causes of deaths	Risk factors	Community	2 years	Mortality
South Africa 1997–2000^32^	92	Infective endocarditis	Cardiac status, status for infection	Hospital	6 months	Mortality
Sudan 1977–1986^99^	101	Type 1 diabetes	Status at diagnosis	Hospital	10 years	Hospitalisation mortality
Sudan 1987–1990^27 100^	327	Type 1 diabetes	Incidence study	Hospital/Community	4–10 years	Incidence
Tanzania 1981–87^38,101-104^	1250	Newly diagnosed diabetes	Risk factors	Hospital	7 years	Mortality complications, trajectories
Tanzania 1986^42^	239	Glucose tolerance	Glycaemia, BP, BMI, lipids	Community	1 year	Change in risk factors, diabetes incidence
Zimbabwe^105^	528	BP	Psychological predictors	Workplace (university)	4 years	Hypertension
Zimbabwe 1971^20^	107	Diabetes mellitus	Status for diabetes	Hospital	6 years	Case fatality
Zimbabwe 1986^106^	75	Rheumatic fever	Heart status	Hospital	1–12 years	Cardiac complications

BP = blood pressure, BMI = body mass index.

**Table 2:**
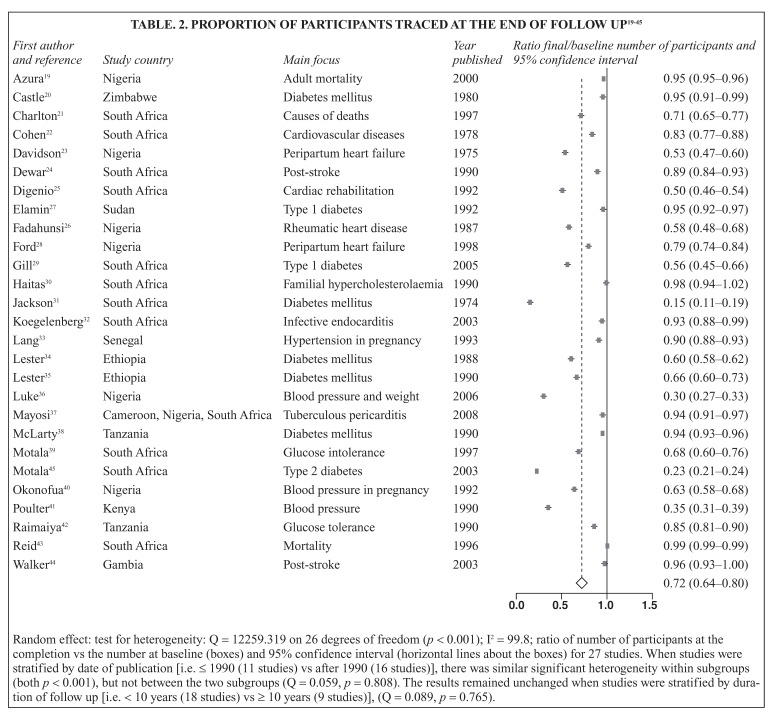


## Cohort with a focus on mortality at baseline or during follow up

*Stroke*: five hospital studies have followed individuals in the post-stroke period for mortality and disabilities.^24^,^44^,^46^,^58^,^61^,^62^ Collectively, these studies have provided follow-up information on about 1 244 individuals over a period of six months to four years.

*Heart failure*: two studies in Nigeria monitored the outcomes of patients with specific types of heart failure: hypertensive and peripartum heart failure. Izuezo and his colleagues^52^ monitored a cohort of 107 patients with hypertensive heart failure for mortality over a 12-month duration. The rate of death was 29%, and 22% of these deaths were recorded within the first three months of follow up. Predictors of death included the duration of diagnosed heart failure, blood pressure variables, age, baseline serum creatinine, and treatment with captopril.

Parry and his collaborators^23^,^28^,^53^-^55^ followed for more than 20 years a cohort of 227 women with peripartum heart failure at baseline in Zaria. The mortality rate in this study was 11% within two to five years of follow up, and 42% at 20 years; the majority being cardiovascular. Predictors of death and changes in the status of heart failure were investigated. However, the techniques used for such analysis failed to accommodate the varying time of occurrence of event between participants, and was unable to provide information beyond differences in the mean levels or prevalence of predictors between subgroups of participants. Parry and his colleagues also described the changing trends of blood pressure variables and the incidence of hypertension in the cohort.

*Rheumatic heart disease, cardiac infections*: three cohort studies with a focus on rheumatic heart disease were identified. A group of 75 patients with acute rheumatic fever was monitored in Zimbabwe for about 12 years for incident cardiac complications and deaths.^106^ The average time to development of chronic valvular disease and heart failure was 2.8 and 5.2 years, respectively, and death was likely to occur in young patients at baseline. Fadahunsi^26^ in Nigeria reported on a cohort of 92 patients with established rheumatic heart disease from the time of their first hospital contact. Retention of participants was low, with only one-third of participants still regularly attending visits after 10 years into the study.

In 1972, a clinical screening was conducted on 12 050 schoolchildren in Soweto, during which 168 children had auscultation signs suggestive of rheumatic valvular disease.^22^ Four years later, 139 of these children were traced and re-examined to monitor changes in their heart sounds in the absence of prophylaxis against rheumatic activity. This re-examination revealed that most of the auscultation abnormalities identified during the first examination were not features of rheumatic heart disease. However, recently, asymptomatic patients without cardiac murmur were found to have echocardiographic abnormalities that were suggestive of rheumatic heart disease.^107^ The clinical and prognostic significance of clinically silent echocardiographic abnormalities of suspected rheumatic heart disease needs to be determined in prospective randomised studies of penicillin prophylaxis.

Two cohort studies followed patients in relation to cardiac infections in SSA. From 1997 to 2000, Koegelenberg in South Africa investigated and followed patients referred for presumptive infective endocarditis.^32^ Of the 92 examined at baseline, 35% had a diagnosis other than infective endocarditis. Seventy-three per cent of those with infective endocarditis had a history or echocardiographic features of rheumatic heart disease. Eighty-one per cent of those without infective endocarditis had underlying rheumatic heart disease. The six-month crude mortality rate was 35.6% in those with a confirmed diagnosis of infective endocarditis, and 12.9% among those without.

Between March and October 2004, a cohort of 185 patients with presumptive pericardial tuberculosis was established from 15 referral hospitals in Cameroon, Nigeria and South Africa.^37^ These patients were observed for a six-month period under specific treatment for major outcomes, including mortality. The overall mortality rate was 26% among 174 patients, with information available on their vital status at the study completion. Using Cox regression models, independent predictors of death during follow up were: a proven non-tuberculosis final diagnosis, the presence of clinical signs of HIV infection, coexistent pulmonary tuberculosis, and older age.^37^ Among survivors, clinical signs of HIV infection at baseline were associated with lower risk of developing a pericardial constriction during follow up.^108^

*Multiple outcomes*: we identified one community-based intervention study, the Coronary Risk Factor Study (CORIS). This multifactorial community intervention programme went on for four years, and a post-study evaluation was conducted 12 years after baseline evaluation. Risk factors were measured at baseline and their trajectories monitored during follow up, together with trend in the incidence of hard cardiovascular outcomes. Several reports, including baseline and four-year reports, and eight years post-intervention reports have been published.^70^-^85^

The Idikan Adult Mortality Study was a prospective community-based study which aimed to provide all-cause and cause-specific mortality data for adult Nigerians.^19^,^56^ The two- and five-year follow-up reports were published. Some predictors of mortality, including cardiovascular risk factors (smoking) have been characterised.^19^ The commonest known cause of death in the five-year report was cardiovascular disease. It was responsible for 43 (18.5%) of all deaths. Another small-scale community study in South Africa monitored a cohort of 200 adults over a two-year period for mortality in relation to the level of physical activity, blood pressure and other risk factors.^21^ In this retrospective cohort of older individuals, serum albumin, diabetes status and waist/hip ratio were predictors of two-year mortality.

## Cohorts of risk factors and trajectories over time or hard outcomes

*Child and adolescent cohorts of CVD and its risk factors*: the ‘Mandela’s children’ cohort is the largest and longest ongoing African initiative regarding child and adolescent health.^88^-^90^ The study started in 1989/1990 with 3 273 newborn infants in Soweto, South Africa.^109^,^110^ It was initially designed for a 10-year follow-up duration and labelled Birth-To-Ten Cohort; this changed to Birth-To-Twenty when the duration was extended to 20 years.^111^ A major contribution to cardiovascular epidemiology has been the study of the trajectories of cardiovascular risk factors in this cohort.^88^,^89^

The Ellisras Longitudinal Study (ELS) is an ongoing study of the growth and health of rural South African children. It was initially designed as a mixed longitudinal study to investigate the growth and nutritional status of rural South African children attending pre-primary and primary school (3–10 years old).^95^ Initial data collection was limited to anthropometric assessment, however from the year 2000, data were also collected on blood pressure variables.^93^,^94^,^96^,^97^ Baseline data provided information on the prevalence of risk factors, particularly obesity^98^ and hypertension.^94^ The follow up will provide additional information regarding changing trends with time.

*Blood pressure variables and control of hypertension*: the Luo Migration Study in Kenya provided a picture of the changing pattern of blood pressure variables with time. Some predictors of these changes were the result of migrating from rural to urban areas.^41^,^49^-^51^ The dropout rate was very high, with only 35% of the original sample available for the final evaluation.

The International Collaborative Study on Hypertension in Blacks (ICSHIB) was a comparative study on hypertension and its determinants in geographically separated black populations.^112^ After a baseline risk-factor survey from 1995 to 1999, a follow-up component was initiated in three participating countries, including Nigeria. The five to seven years of follow up provided information regarding changing patterns of weight and blood pressure.^36^,^59^ The two-year follow-up data have already identified blood pressure as a significant determinant of all-cause mortality, with a 60% greater risk associated with each 20 mmHg higher diastolic blood pressure.^60^

In a cohort of 528 university students in Zimbabwe, Somova assessed some psychological predictors and related them to incident hypertension and trajectory of blood pressure variables during a four-year follow-up period.^105^ In multivariate analysis, these psychological factors were significant predictors of hypertension. Four hospital studies from four countries totalling 4 488 participants have focused on blood pressure changes under treatment or no treatment, incidence of hypertension, and other health effects of higher-than-optimal blood pressures.^33^,^40^,^48^,^91^,^92^ Two of these studies (1 186 participants) were conducted in pregnant women.^33^,^40^

*Glucose exposure and diabetes control*: 10 studies on diabetes mellitus or glucose tolerance status in relation to new onset of diabetes, changes in blood glucose levels, incidence of diabetes complications, and all-cause mortality during follow up were found. Three were community-based studies of incident diabetes according to baseline status for glucose tolerance.^31^,^39^,^42^,^45^,^87^

In the Hindu community study in Tanzania, blood glucose and blood pressure levels improved within four years of follow up, an improvement that investigators ascribed to community action.^42^ Over a longer period of follow up (10 years) of a South African Indian cohort, Motala found a 0.95% annual rate of progression to diabetes.^45^ Two-hour post-load glucose, body mass index and obesity were baseline predictors of incident diabetes in this study. Jackson^31^ earlier had reported on the five-year incidence study of Tamilian Indians first examined in Cape Town in 1965.

In Tanzania, at the diabetes clinic of the Muhimbili Medical Center in Dar es Salaam, McLarty and his colleagues^38^,^101^,^103^ monitored a group of individuals with type 1 and type 2 diabetes from clinical diagnosis between 1981 and 1987, to approximately seven years. The death rate during follow up of the initial cohort of 1 250 individuals was 22%; 24% of these deaths were due to cardiovascular and renal causes. Insulin treatment was strongly associated with death. A sub-cohort of 793 participants was also monitored for the incidence of hypertension.^102^ The change in body mass index was the main predictor of increasing systolic blood pressure. Relating data from this study to the catchment area’s population of the study hospital, the incidence of type 1 diabetes was ascertained.^104^

The biggest hospital cohort of individuals with diabetes was from the Yekatit 12 Hospital diabetes clinic registry in Addis Ababa.^34^,^35^ By 1990, this clinic had registered 1 699 patients first diagnosed with any type of diabetes mellitus after 1969, except for 204 patients who were lost to follow up. Their follow up over varying durations has provided information relating to the incidence of a range of diabetes complications and mortality.^35^ Trajectories of other risk factors such as body mass index and blood pressure variables, as predictors of survival, were alluded to in this study.^47^

Keeton^69^ monitored a cohort of 62 individuals with type 2 diabetes in Cape Town, South Africa for renal outcome over a 12-year period. In this high-risk group at baseline, the death rate during follow up was 79%, with one-third of these deaths being related to chronic renal failure. The study found a correlation between deteriorating kidney function and blood pressure variables. Varying time to event was not accounted for in the data analysis.

*Smoking exposure*: gold miners in South Africa are legally required to have an initial and a yearly medical examination at the Medical Bureau for Occupational Diseases (MBOD). The registers of the MBOD were prospectively utilised in evaluating the risk of occupational diseases and other entities, including cardiovascular disease-related risk factors.^43^,^63^,^67^ A major advantage of this cohort was the completeness of data collected. The contribution of exposure to smoking to the risk of disease has been one of the major focus points of this cohort.^64^-^66^

*Lipid variables, dyslipidaemia and adiposity*: lipid variables were assessed along with other risk factors in some studies. In the Hindu study of glucose tolerance in Dar es Salaam, no significant difference was found between the baseline and four-year average levels of total cholesterol and triglycerides and body mass index.^42^ A cohort of 49 individuals with familial hypercholesterolaemia was followed in South Africa over a 13-year period in relation to the natural history, including cardiovascular outcomes.^30^ CVD accounted for 82% of the 11 deaths registered. Survivors displayed an array of cardiovascular lesions.

*>Non-cardiovascular cohorts with potential cardiovascular application*: the Africa Centre Demographic Information System (ACDIS) cohort was started in 2000 in KwaZulu-Natal, South Africa.^113^ It was established to describe the demographic, social and health impacts of the HIV epidemic in a population going through a health transition, and to monitor the impact of intervention strategies on the epidemic. As of June 2006, 85 855 participants from approximately 11 000 households have been under surveillance. Blood pressure variables, weight and height have been measured for women from 15 to 49 years and men aged 15 to 54 years. Outcomes monitored included death. There is an opportunity within this cohort to relate baseline blood pressure variables and anthropometric measurements to incident all-cause and cause-specific deaths.

## Discussion

The sub-Saharan African region is in health transition as high blood pressure, high cholesterol levels and tobacco usage are already among the top risk factors of the CVD epidemic. Empirical data demonstrate that lifestyle modification and early diagnosis are critical for prevention of CVD. The epidemic poses an enormous socio-economic burden and will cripple the region. Evidence from around the world suggests that major determinants of cardiovascular diseases have been identified. These determinants are consistent across populations and regions and may not need to be ‘rediscovered’ in sub-Saharan Africa.^10^ However, as recognised by other investigators,^114^ a better understanding of their epidemiology in SSA ‘will permit the development of more effective public health interventions to forestall a future epidemic of CVD’.

The present review suggests that the magnitude of the burden of CVD risk factors, their interrelation and how they affect incident CVD in SSA are less well known. The few available studies have several methodological shortcomings, including smaller sample size and accordingly lower statistical power for answering relevant questions. Furthermore, the significant heterogeneity between studies precludes data pooling in the studies or at individual level to increase the statistical power.

A series of reviews on cardiovascular diseases in SSA has recently highlighted the importance of and need for local research on CVDs and how they are currently managed in SSA.^1^,^10^,^11^,^115^,^116^ Current and projected health changes operating in SSA confirm that these countries are going through the earlier and intermediate stages of epidemiological transition.^11^ This provides a unique opportunity to probe some of the unexplained period effects in the epidemic of CVD-associated economic expansion, which may yield some aetiological clues to their environmental determinants. The natural epidemiological experiments created by the high prevalence of some chronic infectious diseases (HIV/AIDS, viral hepatitis, tuberculosis) in SSA provide another unique opportunity for assessing their contribution to the burden of CVD.

Efforts to fill the gaps in knowledge on CVD in SSA must be nested with interventions aimed at translating the current knowledge regarding interventions into practical strategies that will limit the burden of cardiovascular diseases in this population. In the absence of relevant data specific to SSA, evidence derived from other populations has been used to inform cardiovascular disease prevention initiatives in Africa. This uncritical application of recommendations derived from elsewhere to SSA populations may be inappropriate, as discussed elsewhere.^115^

In addition, where in existence, recommendations for cardiovascular disease prevention in SSA have not yet embraced the concept of the global risk approach.^118^ It is well known that the traditional single risk-factor approach to CVD prevention does not capture the mutifactorial nature of CVD, and the continuum of risks associated with many risk factors. This approach leads to the inappropriate assignment of individuals to prevention therapies, and inappropriate health resource allocation, and therefore must be discouraged in SSA.

Limited resources may be a constraint to the adoption of global risk tools, particularly those that include laboratory measurements, such as lipid variables, in their calculation. However, the WHO and other investigators have developed non-laboratory versions of such tools.^119^,^120^ Although these would require some recalibration to adjust their performance to the SSA setting, the use of such tools should be encouraged in SSA, alongside other CVD prevention strategies. Ultimately, when local cohort data become available, global risk tools specific to the SSA population should be developed, given that recalibration may not be successful in all circumstances.

## Study limitations

The present review has some limitations that are worth mentioning. These include our inability to capture data available at only the country level, such as theses and health reports. By restricting the review to those articles with an abstract in English, indexed to two major databases, we have possibly missed some studies published in other languages and probably not indexed. This would be the case particularly for publications in French, the official language of a number of African countries. We are not aware of a dedicated online scientific database for these countries.

We did not include in our search strategies terms relating to early antecedents of CVD, such as obesity, physical activity or unhealthy eating habits, since we felt that their effects on CVD are mediated by the other factors accounted for in the review (those are diabetes mellitus, hypertension and dyslipidaemia). Similarly, scoring tools such as ‘metabolic syndrome’ and ‘absolute risk tools’ were not included in the search terms, again as these refer to the combination of those risk factors already included in the search terms, and their uptake in SSA remains very limited.

## Conclusions

Prospective cohort studies are needed to inform and update our knowledge regarding the epidemiology of cardiovascular diseases, and how this changes with time as a result of the natural history and implementation of preventative strategies. The case for cohort studies on non-communicable diseases in general in Africa is available in more detail from Holmes *et al*.^121^ Interaction with investigators in SSA suggests that two limited-scale longitudinal studies on CVDs are in the planning stage in SSA.

The Abuja Heart Study, which started in 2008, will follow 3 000 individuals in Nigeria for five years for CVD outcomes in relation to socio-economic status.^122^ The Prospective Urban and Rural Epidemiological Study (PURE) is a multinational observational study of the effects of societal changes on the burden of chronic diseases, including CVDs.^123^ Three SSA countries (Tanzania, Zimbabwe and South Africa) are involved in this study. Collectively, these studies will provide future useful, although still limited knowledge relating the burden of CVD in SSA.

There is probably no requirement for cohort studies in each SSA country, for both scientific and feasibility reasons. Filling the gaps and providing definitive evidence on CVD in SSA may require only continuous follow up of a diversified population of sufficiently large size. This will provide for the investigation of known and putative risk factors, including genetic predisposition, their interrelationships, and changing patterns with time. Additionally, it will allow for the quantification of the burden of CVD within the context of competing health risks, through exhaustive baseline assessments, including the establishment of bio-repositories for future investigations. With such a community study set up, additional efforts could consist of establishing multi-centre registers in major SSA hospitals to monitor the incidence, management and outcomes of patients with specific profiles, and regularly update prevention strategies.
